# A Novel Approach for Fully Automatic Intra-Tumor Segmentation With 3D U-Net Architecture for Gliomas

**DOI:** 10.3389/fncom.2020.00010

**Published:** 2020-02-18

**Authors:** Ujjwal Baid, Sanjay Talbar, Swapnil Rane, Sudeep Gupta, Meenakshi H. Thakur, Aliasgar Moiyadi, Nilesh Sable, Mayuresh Akolkar, Abhishek Mahajan

**Affiliations:** ^1^Department of Electronics and Telecommunication Engineering, Shri Guru Gobind Singhji Institute of Engineering and Technology, Nanded, India; ^2^Department of Pathology, Tata Memorial Centre, Tata Memorial Hospital, Mumbai, India; ^3^Department of Medical Oncology, Tata Memorial Centre, Tata Memorial Hospital, Mumbai, India; ^4^Department of Radiodiagnosis and Imaging, Tata Memorial Centre, Tata Memorial Hospital, Mumbai, India; ^5^Department of Neurosurgery Services, Tata Memorial Centre, Tata Memorial Hospital, Mumbai, India

**Keywords:** glioma, intra-tumor segmentation, convolutional neural network, deep learning, 3D U-Net

## Abstract

**Purpose:** Gliomas are the most common primary brain malignancies, with varying degrees of aggressiveness and prognosis. Understanding of tumor biology and intra-tumor heterogeneity is necessary for planning personalized therapy and predicting response to therapy. Accurate tumoral and intra-tumoral segmentation on MRI is the first step toward understanding the tumor biology through computational methods. The purpose of this study was to design a segmentation algorithm and evaluate its performance on pre-treatment brain MRIs obtained from patients with gliomas.

**Materials and Methods:** In this study, we have designed a novel 3D U-Net architecture that segments various radiologically identifiable sub-regions like edema, enhancing tumor, and necrosis. Weighted patch extraction scheme from the tumor border regions is proposed to address the problem of class imbalance between tumor and non-tumorous patches. The architecture consists of a contracting path to capture context and the symmetric expanding path that enables precise localization. The Deep Convolutional Neural Network (DCNN) based architecture is trained on 285 patients, validated on 66 patients and tested on 191 patients with Glioma from Brain Tumor Segmentation (BraTS) 2018 challenge dataset. Three dimensional patches are extracted from multi-channel BraTS training dataset to train 3D U-Net architecture. The efficacy of the proposed approach is also tested on an independent dataset of 40 patients with High Grade Glioma from our tertiary cancer center. Segmentation results are assessed in terms of Dice Score, Sensitivity, Specificity, and Hausdorff 95 distance (ITCN intra-tumoral classification network).

**Result:** Our proposed architecture achieved Dice scores of 0.88, 0.83, and 0.75 for the whole tumor, tumor core and enhancing tumor, respectively, on BraTS validation dataset and 0.85, 0.77, 0.67 on test dataset. The results were similar on the independent patients' dataset from our hospital, achieving Dice scores of 0.92, 0.90, and 0.81 for the whole tumor, tumor core and enhancing tumor, respectively.

**Conclusion:** The results of this study show the potential of patch-based 3D U-Net for the accurate intra-tumor segmentation. From experiments, it is observed that the weighted patch-based segmentation approach gives comparable performance with the pixel-based approach when there is a thin boundary between tumor subparts.

## Introduction

According to the Central Brain Tumor Registry of the United States (CBTRUS), 86,970 new cases of primary malignant and non-malignant brain tumors are expected to be diagnosed in the United States in 2019^1^. An estimated 16,830 deaths attributed to primary malignant brain tumors in the US in 2018. Gliomas are the most frequent primary brain tumors in adults and account for 70% of adult malignant primary brain tumors. Glioma arises from glial cells and infiltrates the surrounding tissues such as white matter fiber tracts with very rapid growth (Menze et al., 2015). Patients diagnosed with Glioblastoma tumors have an average survival time of 14 months (Louis et al., 2007).

Accurate segmentation of brain tumor tissues from Brain MR images is of profound importance in many clinical applications such as surgical planning and image-guided interventions (Mahajan et al., 2015). Manual tracing and detection of organs and tumor structure from medical images is considered as one of the preliminary steps in disease diagnosis, treatment planning, and monitoring tumor growth with follow-up evaluation (Udupa and Saha, 2003). In a clinical setup, this time-consuming process is carried out by radiologists, however, this approach becomes impractical when the number of patients increases. This presents an unmet need for automated segmentation methods (He et al., 2019; Vaidya et al., 2019).

**Figure 1:**
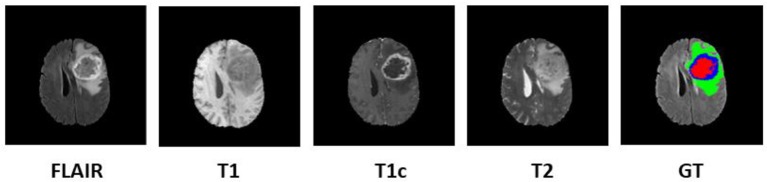
Multi-modal data with four channels provided in BraTS 2018 challenge dataset along with Ground Truth (GT). Sub tumor parts are represented as—Edema: Green, Enhancing tumor: Blue, Necrosis: Red.

In order to diagnose abnormality in brain tissues, various radio imaging techniques like Magnetic Resonance Imaging (MRI), Computed Tomography (CT), and Positron Emission Tomography (PET) are used. Over the last few decades, because of the better soft-tissue contrast, MRI is widely used to assess the brain tissues in clinical practices. Unlike X-rays or CT scans the intensity signature is variable in MRI due to various acquisition protocols. The same tumor cells follow different intensity distribution when acquired with different scanners with varying field strength, voxel resolution, and field of view. More accurate composite marking of the tumor regions can be achieved with four distinct MR sequences like T1, T2, T1 post-contrast (T1ce), and Fluid Attenuated Inversion Recovery (FLAIR). Intra-tumor parts for these four MR sequences with varying intensity can be visualized in Figure 1.

Different heterogeneous intra-tumor regions like edema, active tumor, and necrotic regions are present in Glial brain tumors. Intra-tumor segmentation in the brain has been challenging task because of its several characteristics such as non-rigid and complex appearance, variation in size, and position of tumor from patient to patient. Poor delineation of the intra-tumor parts in multi-modal MRI data as well as similar textural properties of the pathology with healthy tissues make the segmentation task more prone to error. It has been observed that even expert raters show significant variations in case of poor intensity gradients between tumor and rest of the healthy brain tissues. Though several algorithms have been proposed over the decades to address this task, most have shortcomings limiting their utility in routine clinical practice.

The aim of this study is to design a fully automated brain tumor segmentation algorithm which will accurately segment the tumors and act as an assistive tool for radiologists for exact tumor quantification. We have proposed a fully automatic brain tumor segmentation with 3D U-Net architecture based on Deep Convolutional Neural Networks. An efficient weighted patch extraction method along with a unique number of feature maps at each level of 3D U-Net is proposed for accurate intra-tumor segmentation.

We briefly review conventional and recent methods for brain tumor segmentation algorithms available in the literature. Further, BraTS challenge database along with local dataset from our hospital and proposed methodology for tumor segmentation is described. This is followed by experimental results, quantitative as well as a qualitative evaluation of the results and comparison with other methods. Finally, we conclude the manuscript with future directions.

## Literature Review

As mentioned by Menze et al. there is a linear increase in the tumor imaging literature over the past 30 years and over 25% of the publications aimed at “automated” tumor segmentation. Segmentation of the glial tumors is the primary focus in most of the existing methods and very few methods targeted for specific glioma subtype or meningioma (Bauer et al., 2013). The brain tumor segmentation methods are broadly classified into two categories based on generative probabilistic based models and discriminative approaches. Generative probabilistic based approaches detect abnormal regions by comparing it with explicit models of anatomy and outlier detection. On the other hand, discriminative models learn from feature-based differences between normal tissues and tumor tissues.

Generative models aim at finding the outliers between *a-priori* model of a healthy brain (atlas) and the abnormal regions. This uses the prior information of tumor appearance and spatial distribution of the brain tissues and these methods exhibit good generalization to an unseen database (Prastawa et al., 2004). Cordier et al. (2016) proposed a fully automatic patch-based approach for Glioma segmentation with the multi-atlas voting technique with less prior learning to avoid overfitting. The major drawback of these approaches is that it relies heavily on domain-specific prior knowledge and accurate multi-modal image registration. Because of the presence of large abnormalities and resection cavities in the brain, the multimodal registration miserably fails which lead to inaccurate segmentation in generative models.

Discriminative models directly learn from hand-designed features calculated on lesions and other brain tissues. This is carried out on large datasets to avoid the effect of imaging artifacts, intensity, and shape variations. In these approaches, various dense, and voxelwise features are extracted from MR images and fed into the classification algorithms like decision trees and support vector machines (Criminisi and Shotton, 2013). Demirhan et al. (2015) employed a method based on wavelets and Self-Organizing Maps (SOM) to segment intra-tumor parts along with healthy brain tissues. The drawback of these approaches is that, since the segmentation highly relies on the intensity, texture features etc. of the training data, segmentation is specific to the MRI images acquired with the same imaging protocol as of the training dataset.

Balafar et al. reviewed brain tumor segmentation methods and further classified them into four categories as Threshold-based, Region-based, Pixel classification based, and Model-based techniques with pros and cons over each other (Balafar et al., 2010). Many approaches to brain tumor segmentation have been implemented over decades but there is no winning theory.

Recent methods based on Deep Convolutional Neural Networks have outperformed all traditional machine learning methods in various domains like medical image segmentation, image classification, object detection, and tracking etc. (Smistad et al., 2015) and are currently considered to be art in biomedical image segmentation (Moeskops et al., 2016; Pereira et al., 2016; Havaei et al., 2017). The computational power of GPUs has enabled researchers to design deep neural network models with convolutional layers which are computationally expensive (Eklund et al., 2013; Eminaga et al., 2018; Lee et al., 2018; Leyh-Bannurah et al., 2018).

Pereira et al. (2016) proposed an automatic segmentation method using Convolutional Neural Networks by exploring smal 3 × 3 kernels. 2D patches were extracted from four MR channels of size 33×33 for training the network. Ronneberger et al. (2015) segmented the neuronal structures in electron microscopic stacks with 2D U-Net architecture trained on transmitted light microscopy images with augmentation of the training data by geometrical image transformations. Kamnitsas et al. (2017) proposed dual pathway architecture with dense training scheme to incorporate both local and larger contextual information. The architecture processed the input images at multiple scales simultaneously. False positives in the segmentation maps were minimized using Conditional Random Forests (CRF).

Inspired from the above literature, we developed a novel Deep Convolutional Neural Network-based 3D U-Net model with a unique number of feature maps. Various heterogeneous histologic sub-regions like peritumoral edema, enhancing tumor, and necrosis were accurately segmented in spite of thin and/or fuzzy boundaries between intra-tumor parts with this proposed architecture.

## Patients and Method

We focused our experimental analysis on MICCAI (Medical Image Computing and Computer-Assisted Intervention) Brain Tumor Segmentation (BraTS) 2018 challenge (Bakas et al., 2019). BraTS dataset consisted of multi-institutional routine clinically acquired pre-operative multimodal MRI scans of High Grade Glioma i.e., Glioblastoma (GBM/HGG) and Lower Grade Glioma (LGG), with a pathologically confirmed diagnosis. In the challenge, MR data of 285 patients for training, 66 for validation and 191 patients were provided in the test dataset. The MR data was acquired with different imaging clinical protocols and various MR scanners with 19 distinct institutions (Bakas et al., 2017a,b). Each patient data was provided with FLAIR, T1, T2, T1 post-contrast MR volume of size 240×240×155 which were resampled to 1 mm × 1 mm × 1 mm resolution. Segmentation labels as edema, enhancing tumor, and necrosis were annotated for all patients by one to four radiologists as shown in Figure 1. These segmented labels were also verified by expert neuro-radiologists. The main task of BraTS 2018 challenge was to auto-segment the tumor into its three constituent regions viz.

1. Enhancing tumor region (ET)
2. Tumor Core (TC) which entails the ET, necrotic (fluid-filled) and the non-enhancing (solid) parts
3. Whole tumor (WT) which includes all intra-tumor parts along with Edema.

Apart from BraTS 2018 dataset, the proposed method was also tested on 40 pre-treatment multimodal MRI patient datasets of Glioblastoma (GBM) from our hospital. MR data of four channels as FLAIR, T1, T2, and T1 post contrast was collected for the study. The acquisition protocol is provided in Supplementary Material. The local dataset was explicitly used for the purpose of testing only. This dataset was also skull-stripped and resampled to 1 mm × 1 mm × 1 mm resolution. This dataset was annotated by the expert radiologists from our hospital with the same protocol which was defined to annotate BraTS challenge dataset (Menze et al., 2015).

### Pre-processing

The input data for the segmentation algorithm were skull stripped, normalized, and co-registered to an anatomical template (Smith, 2002). In order to normalize the signal intensities between the BraTS and our hospital datasets, bias field correction was performed with N4ITK tool (Tustison et al., 2010). Further, MR data of each channel was normalized by subtracting the channel mean and dividing by the variance i.e., zero mean and unit variance.

### Patch Extraction

Tumor sub-region distribution in BraTS training data was highly imbalanced. Further, 98% pixels of the dataset belonged to either healthy brain tissues or background and hence the model was prone to overfit on non-tumor tissues only. The problem was exaggerated when the prediction was made based on center pixel class of the patch. Hence, precise patch selection from the input data for training is of extreme importance. To overcome this problem, we adopted a novel 3D patch-based approach for training with weighted sampling. Zhou et al. (2019) reviewed 2D and 3D patch extraction methods along with several types of loss functions. The main approaches included resampling the data space as: under-sampling the negative class or up-sampling the negative class and SMOTE (Synthetic Minority Over-sampling Technique) generating synthetic samples. The methods discussed includes patch extraction 50% probability being cantered either on the lesion or healthy voxel. Also, all training patches centered on a lesion voxel. AlBadawy et al. (2018) discussed impact of cross institutional training and testing for segmentation of brain tumors. In this study patches of 33^*^33 were extracted on T1, T1ce, and FLAIR modality. Our proposed approach differs with this approach in terms of dimension of patch size as 64^*^64^*^64. It is well-known fact that T2 modality is widely used to distinguish tumor core boundary with rest of the tumor and hence we included T2 channel as well along with the other three MR channels to incorporate more information during training.

**Figure 2:**
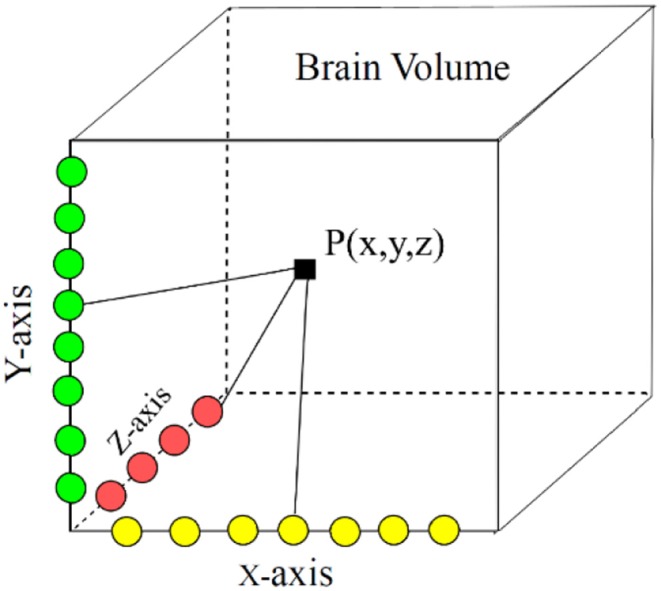
Patch center localization by randomly selecting x, y, z coordinates in brain volume.

**Figure 3:**
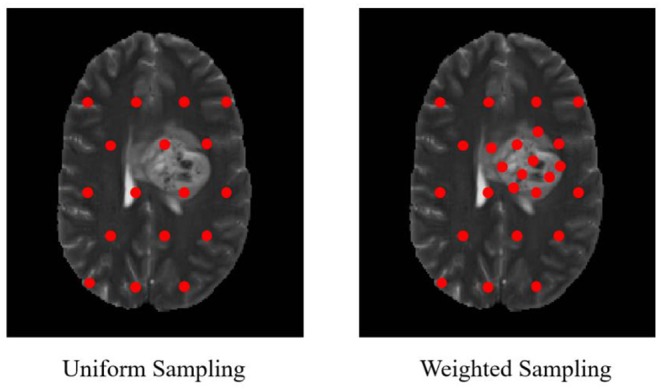
Uniform sampling and weighted sampling for patch extraction.

In our proposed approach, 3D patches were extracted from all the four modalities so that the network can be trained on a distinct intensity signature of intra-tumor tissues in each modality. For this, we considered the equidistant seed points in X, Y, and Z directions of the MR data as shown in Figure 2. A 3D patch of size 64 × 64 × 64 voxels was considered around each seed point. In the next step, potential patches which had brain area more than 60% of the total patch were only considered for the training to minimize the chances of overfitting of the model to the background pixels. It was observed that the model was misclassifying the pixels on tumor boundary to healthy brain tissues. A similar problem occurred when tumors were present on the boundary of the brain, with pixels being classified to background. To address this, some patches were explicitly extracted on the boundary of the tumor with weighted sampling as shown in Figure 3. The boundary locations of the WT is considered as the tumor boundary to extract the additional patches. This is done with find_boundaries() function available in segmentation module in popular skimage library. Randomly 30% boundary locations are selected for these extra patch extractions. Since, there is high class imbalance in tumor tissues and healthy tissues, this additional patch extraction does not impact on the performance of model like biased training or overfitting. These additional patches were added to the training patch dataset so that model could be trained in a better way to distinguish thin boundaries of the tumor with the rest of the brain or background. This weighted patch extraction pipeline is fully automatic i.e., without any manual intervention. These 3D patches from all the four channels were concatenated together and given as input to the first layer of the model along with corresponding ground truth during training. During testing as well the non-overlapping patches of size 64 × 64 × 64 were extracted and final output volume is generated by concatenating all these predicted patches to get single 240 × 240 × 155 volume.

### Proposed 3D U-Net Architecture

Conventional U-Net architecture consists of a bunch of basic layers such as convolutional layers, down-sampling and upsampling layers etc. Several variants of the 2D and 3D U-Net architectures are available in the recent literature which mainly differ in respect to the choice of hyperparameters viz. depth of U-Net, number of feature maps, kernel size etc. Selection of these hyperparameters along with accurate region input is of utmost importance for accurate training of the model. The novelty of our proposed approach lies in the weighted patch extraction scheme from the edges of the tumor and designing the structure of 3D U-net with less number of levels and an increased number of filters at each level. Although several deeper U-Net architectures are proposed for segmentation task, we restricted our network to three levels. This reduced the number of trainable parameters but also avoided the bottleneck problem caused due to smaller patch size.

**Figure 4:**
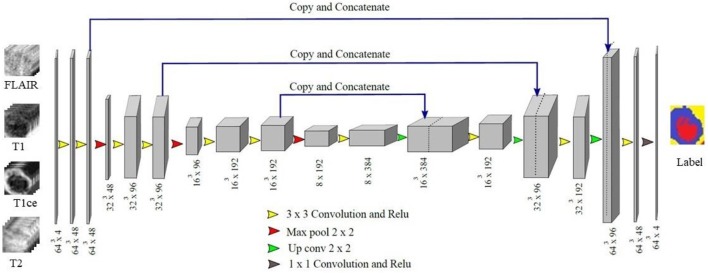
Proposed 3D U-Net Architecture. Voxels from all four MR channels were given input to the first layer of the model. The predicted labels were compared with the Ground truth to calculate Dice loss.

In proposed 3D U-Net architecture, from the first level to third level 48, 96, and 192 feature maps were present at each subsequent level in down-sampling and up-sampling layers as shown in Figure 4. The proposed architecture consisted of a contracting path to capture context and a symmetric expanding path that enables precise localization. At the first layer four 64 × 64 × 64 multichannel MR volume data was given as input for training along with the corresponding ground truth. The number of features maps increased in the subsequent layers to learn the deep tumor features. These were followed by ReLU activation function and the features were down-sampled in encoding layer. Similarly, in decoding layer after convolution layers and ReLU activation function, features maps were up-sampled by a factor of 2. Features maps from encoding layers were concatenated to the corresponding decoding layer in the architecture. In contrast to conventional U-Net, all the feature maps were zero-padded to keep the same output dimensions for all convolutional layers. Finally, four output maps were generated with 1 × 1 convolutional layer corresponding to non-tumor tissue, edema, necrosis, and enhancing tumor. Each voxel of these four output maps corresponds to the probability of each voxel belonging to the particular class. The final prediction was generated by selecting the label with maximum probability from these four label maps. At the output layer, the segmentation map predicted by the model was compared with the corresponding ground truth and the error was backpropagated in the intermediate 3D U-Net layers.

In our implementation, the learning rate (α) was initialized to 0.001 and remained unchanged till 60 epochs. Since, after 60 epochs the Dice loss stopped improving, we decreased it linearly by a factor of 10^−1^ which avoided convergence of the model to local minima. The model is trained for 100 epochs since beyond that there was no significant improvement in the Dice loss and hence the training was terminated. Dropout with ratio 0.25 was added during training to avoid overfitting. The architecture was trained with a batch size of 8. Further, for better optimization a momentum strategy was included in the implementation. This used a temporally averaged gradient to damp the optimization velocity.

### Post-processing

False positives in the segmentation output within the brain region were minimized with 3D Connected Component Analysis with the largest connected component being retained in each predicted volume. Similarly, false positives from the background were eliminated using a binary brain mask generated from brain volume and overlaid on the segmentation output with a logical AND operation. This improved the accuracy of the segmentation significantly for tumors present on the boundaries of the brain. There are some limitations of 3D connected component analysis as post-processing method where bifocal tumors are present that too in distinct brain lobes.

### Implementation Details

The proposed architecture was implemented using Tensorflow library which supported the use of GPUs (Agarwal et al., 2015). GPU implementation greatly accelerated the implementation of deep learning algorithms. The approximate time to train the model was 48 h on 16 GB NVIDIA P100 GPU using cuDNN v5.0 and CUDA 8.0 with 128 GB RAM. The prediction on validation data took <60 s for a single patient with four MR channels data, each of dimension 240 × 240 × 155.

## Results and Discussion

**Figure 5:**
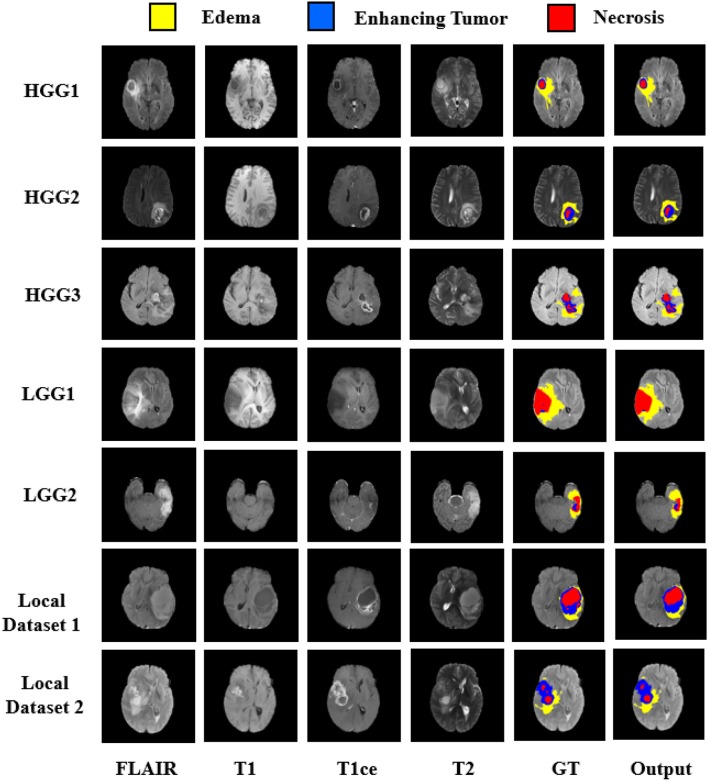
Segmentation results on BraTS 2018 challenge dataset on High Grade Glioma (HGG) and Low Grade Glioma (LGG). In each row from left to right—FLAIR, T1, T2, T1ce, Ground Truth (GT), and predicted output. Segmented Edema, Enhancing Tumor and Necrosis shown with Yellow, Blue, and Red colors, respectively.

The quantitative evaluation of the proposed model was done on BraTS 2018 challenge dataset and also on an independent dataset of GBMs from our hospital. The BraTS dataset comprised of three data sub-sets, viz. training, validation, and test dataset. No ground truths were provided for validation and test dataset. The representative results on BraTS challenge dataset are shown in Figure 5 with High Grade Glioma (HGG) and Low Grade Glioma (LGG). Edema, Enhancing Tumor, and Tumor Core segmented by our approach are shown with Yellow, Blue, and Red colors, respectively.

### Quantitative Performance Evaluation

Performance evaluation was done based on Dice Score, Sensitivity, Specificity, and Hausdorff 95 distance. These evaluation matrices are measures of voxel-wise overlap of the segmented regions (CBICA Image Processing Portal^2^; Taha and Hanbury, 2015). The Dice score normalizes the number of true positives to the average size of the two segmented areas. It is identical to the F score (the harmonic mean of the precision-recall curve) and can be transformed monotonously to the Jaccard score. For the tumor regions Dice Score, Sensitivity (True positive rate), and Specificity (True negative rate) were computed as shown in Equations (1)–(3).

(1) $$\begin{array}{l} Dice\left(P,T\right)\;=\frac{2*|P_{1}\cap T_{1}|}{\left(\left|P_{1}|+|T_{1}\right|\right)} \end{array}$$

(2) $$\begin{array}{l} Sensitivity\left(P,T\right)\;=\frac{\left|P_{1}\cap T_{1}\right|}{\left(\left|T_{1}\right|\right)} \end{array}$$

(3) $$\begin{array}{l} Specificity\left(P,T\right)\;=\frac{\left|P_{0}\cap T_{0}\right|}{\left(\left|T_{0}\right|\right)} \end{array}$$

**Figure 6:**
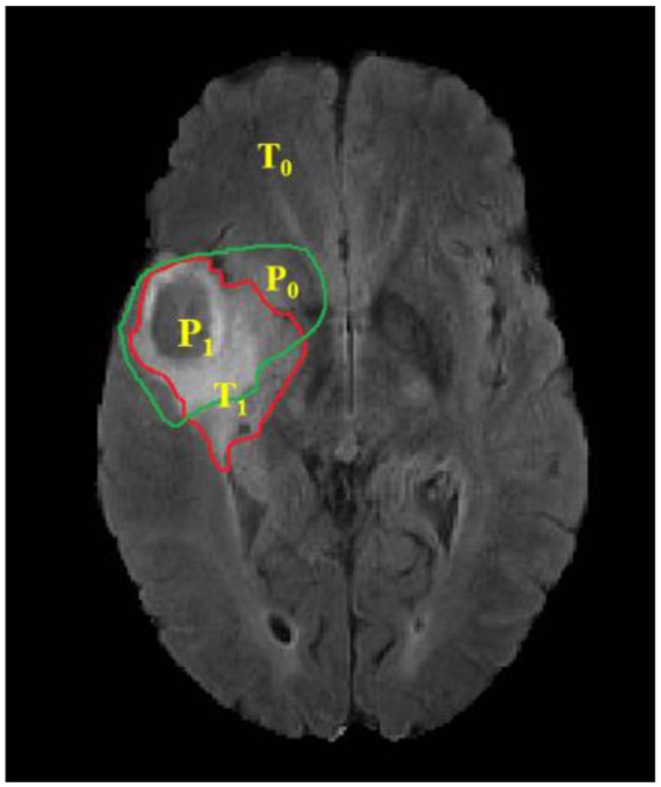
Red contour: ground truth, green contour: predicted segmentation. Notation T is to denote ground truth and P to the predicted segmentation output.

**Figure 7:**
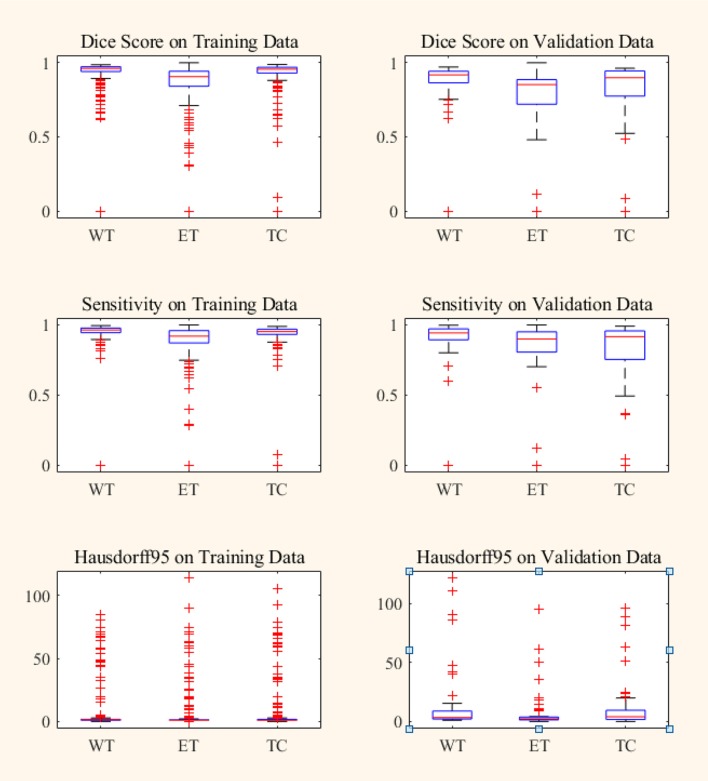
Box plot of Dice score, Sensitivity and Hausdorff 95 distance on BraTS18 training and validation data. Red line within box plot is the median of the corresponding data. ET, enhancing tumor; WT, whole tumor; TC, tumor core.

Where, P represents the model prediction and T represents the Ground Truth labels. T_1_ and T_0_ are the subset of voxels predicted as positive and negatives for tumor region and similar for P_0_ and P_1_ as shown in Figure 6. The Hausdorff 95 distance is the 95th quartile of the maximum overall surface distance between predicted surface and ground truth surface. Hausdorff 95 overcomes the problem of high sensitivity of the Hausdorff measure to small outlying sub-regions from both P1 and T1 (Taha and Hanbury, 2015). Specificity was also calculated and was noted to be >99% in all the cases. Mean, median, standard deviation, 25 quartile, and 75 quartile were also computed for all the patients in the dataset. The BraTS challenge organizers had provided online evaluation system for all the training, validation, and test cases from the BraTS dataset (CBICA Image Processing Portal). The evaluation metrics were calculated by us for the in-house cases (Table 1 and Figure 7).

**Table 1:** Experimental results on BraTS 18 challenge training, testing, and validation dataset.

**Datasets**	**Evaluation parameters**	**Dice**			**Hausdorff 95**		
		**ET**	**WT**	**TC**	**ET**	**WT**	**TC**
BraTS18 Training (285 patients)	Mean	0.8202	0.9324	0.9198	7.0750	11.0278	11.0985
	SD	0.2746	0.1057	0.1327	21.2342	27.9139	29.4150
	Median	0.9062	0.9614	0.9565	1.0000	1.4142	1.0000
	25 Quartile	0.8422	0.9406	0.9303	1.0000	1.0000	1.0000
	75 Quartile	0.9422	0.9728	0.9687	1.4142	1.7320	2.0000
BraTS18 Validation (66 patients)	Mean	0.7480	0.8780	0.8267	7.2951	16.8157	11.2021
	SD	0.2659	0.1346	0.1828	15.7042	30.2509	20.2365
	Median	0.8527	0.9180	0.8985	2.2360	3.3131	4.3589
	25 Quartile	0.7325	0.8665	0.7771	1.4142	2.0000	2.0000
	75 Quartile	0.8853	0.9420	0.9444	3.9354	8.4183	9.4868
BraTS18 Testing (191 patients)	Mean	0.6677	0.8475	0.7688	9.0554	17.2184	14.5728
	SD	0.3120	0.1699	0.2786	19.8975	28.9190	26.1504
	Median	0.8013	0.9050	0.8946	2.2360	3.4641	3.3166
	25 Quartile	0.6557	0.8336	0.7519	1.4142	2.2360	2.0000
	75 Quartile	0.8657	0.9404	0.9328	3.6055	9.4604	8.4844
Our patient dataset (40 patients)	Mean	0.8134	0.9235	0.9012	6.0863	8.1789	9.8647

*ET, enhancing tumor; WT, whole tumor; TC, tumor core; SD, standard deviation*.

In BraTS 2018 training dataset, the mean Dice score for ET, WT, and TC was 0.80, 0.93, and 0.91, respectively. The model predicted ET, WT, and TC with Dice score of 0.75, 0.88, and 0.83 for validation dataset. Comparison of our approach with other methods participated in the BraTS challenge is given in Table 2. From Table 2, it can be observed that our approach achieved better segmentation accuracy in terms of Dice Score over other methods available in the literature. We tested the proposed architecture on 40 patients from our hospital and achieved Dice Score 0.81, 0.92, and 0.90 for ET, WT, and TC, respectively.

**Table 2:** Comparison of proposed architecture with other segmentation methods who participated in BraTS 2018 challenge.

**BraTS18 datasets**	**References**	**Dice**			**Hausdorff 95**		
		**ET**	**WT**	**TC**	**ET**	**WT**	**TC**
Validation	Cabezas et al., 2018	0.7403	0.8892	0.7200	5.3035	6.9563	11.9238
	Chen et al., 2018	0.7334	0.8878	0.8078	4.6426	5.50541	8.14015
	Fang and He, 2018	0.7200	0.8560	0.7260	5.7000	7.5000	9.5000
	Gates et al., 2018	0.6783	0.8055	0.6852	14.5229	14.4150	20.0174
	Hu et al., 2018	0.6100	0.8300	0.7300	41.4800	47.2300	41.1400
	Myronenko, 2019	0.8233	0.9100	0.8668	3.9257	4.5160	6.8545
	Isensee et al., 2018	0.8087	0.9126	0.8634	2.41	4.27	6.52
	Mehta and Tal, 2019	0.7880	0.9090	0.825	3.520	4.923	8.316
	Lefkovits et al., 2018	0.7190	0.8730	0.6890	7.3040	7.0680	12.6630
	Proposed 3D U-Net	0.7480	0.8780	0.8267	7.2951	12.9486	11.2021

*ET, enhancing tumor; WT, whole tumor; TC, tumor core*.

The proposed approach outperformed over other U-net based deep learning approaches available in the literature as shown in Table 2 for training and validation dataset. Since the performance of other methods on test dataset are not available publically and hence not included in the comparison. As different tumor parts appear with distinct intensities in FLAIR, T1, T2, and T1ce modalities, we extracted 3D patches from all the four modalities which resulted in better training for intra-tumor segmentation. Also, we resolved the problems resulting due to focusing on the center pixels of a patch as has been the norm in previous approaches (Pereira et al., 2016) which results in high misclassification due to severe class imbalance in the patches. We instead have merged the four segmentation label maps corresponding to enhancing tumor, necrosis, edema, and background predicted at the output layer, to generate a single segmentation map.

High class imbalance is also intrinsic to most imaging datasets. Around 98.88% pixels belonged to background/healthy class while an average of 0.64, 0.20, and 0.23% pixels belonged to Edema, enhancing tumor and necrosis, respectively. Training of the model with this class imbalance would result in overfitting to the healthy class leading to misclassification of necrotic pixels to healthy pixels. This problem was overcome by weighted sampling and augmenting the data for under-represented regions. Patches from the boundary region of the tumor were added explicitly for better training of the model with weighted patch extraction. All these steps increased the segmentation accuracy at the tumor boundaries.

In patch-based training approaches, larger patches require more max-pooling layers which minimize the localization accuracy. Contrarily, training with small patches allows the network to see only little context. Hence, a classifier output that takes into account the features from multiple layers is considered. This leads to better localization with the use of context. We experimented with various patch extraction size and schemes along with variations in encoding and decoding layers in terms of number and dimension of the Conv-filters. We finalized various hyperparameters like the number of Conv layers, feature maps, activation function, loss function, patch size, learning rate, etc. by extensive experimentation on validation dataset. We evaluated the performance of the model on online evaluation portal for validation dataset and the hyperparameters for which best validation Dice score is achieved are finalized. Three encoding and three decoding layers with 48, 96, and 192 feature maps with ReLU activation function is used in the model with training on patch size of 64 × 64 × 64. The weights of the proposed model are updated according to Dice loss. Some notable variations and performance are provided in Supplementary Table 2.

Box plot for all the patients in BraTS training and validation dataset are shown in Figure 7. It can be observed that the median value is much higher than the mean value in terms of Dice Score. Theoretically, Dice Score ranges from minimum 0 to maximum 1. From the box plots, it can be observed that the Dice scores of two cases for enhancing tumor and tumor core segmentation results are very close to 0 and for the whole tumor is below 0.5 in a few cases. These regions failed to segment accurately because of the high deviation in characteristics in training and validation dataset. This problem can be overcome by increasing the training data with inter-patient variations.

### Grading of Segmentation by Neuroradiologist

The segmentation results on the in-house testing dataset were further evaluated by an in-house expert radiologist (AM) on a scale of 0–5. Score 0 referred to the poor segmentation and 5 for the most accurate delineating of the tumor parts from the healthy tissues. The subjective score for almost all segmented images was found acceptable by the radiologists. However, in a few cases with large necrotic tumor cavity, the proposed algorithm failed to accurately segment the tumor parts. We further investigated the problem and found that such cases were not present in the BraTS challenge training dataset on which proposed architecture was trained and this can be addressed by increasing the training dataset with patients belonging such type of tumor parts. We achieved average 4.1 and median 4 score by the expert Neuroradiologist. The details are provided in Supplementary Table 1. Since, BraTS validation and dataset comprised of the scans from multiple institution with varying protocols the performance on them is comparatively poor. Also, it was observed that on online evaluation portal even if you predict a single pixel for the sub-tumor part which is not present in the patient scan, the Dice score for the corresponding case is zero which reduces the mean Dice score on complete dataset. MR data of all the patients from our in-house dataset was with all the tumor subparts and hence there were no cases for which the Dice score as zero.

## Conclusion

In this paper, we presented fully automatic brain tumor segmentation with a novel 3D U-Net architecture based on Deep Convolutional Neural Networks. An efficient weighted patch extraction method along with a unique number of feature maps at each level of 3D U-Net is proposed for accurate intra-tumor segmentation. The performance of the proposed algorithm is evaluated on BraTS 2018 dataset as well as on the dataset from the local hospital. We considered different training schemes with variable patch sizes, data augmentation methods, activation functions, loss functions, and optimizers. Nowadays, adversarial networks are outperforming state of the art methods for semantic segmentation in several Computer Vision tasks. This can be further inverstigated to improve the segmentation in medical images. The work can also be extended for prediction of overall survival prediction of the patient with the radiomic features computed on the predicted tumor.

## Data Availability Statement

Publicly available datasets were analyzed in this study. This data can be found here: https://www.med.upenn.edu/sbia/brats2018/data.html.

## Ethics Statement

The studies involving human participants were reviewed and approved by the Multimodal Brain Tumor Segmentation Challenge 2018. As this study was carried out retrospectively on pre-existing data, written informed consent for participation was not required in accordance with the national legislation and the institutional requirements.

## Author Contributions

UB, AMa, and SR conducted the experiment. All the authors contributed to writing the manuscript and are responsible.

### Conflict of Interest

The authors declare that the research was conducted in the absence of any commercial or financial relationships that could be construed as a potential conflict of interest.

## Footnotes

^1^Available: http://www.cbtrus.org/www.cbtrus.org/factsheet/factsheet.html

^2^CBICA Image Processing Portal. Available: https://ipp.cbica.upenn.edu/
