# Supplementary material for: A Novel Approach for Fully Automatic Intra-Tumor Segmentation With 3D U-Net Architecture for Gliomas
Source: Front Comput Neurosci. 2020 Feb 18;14:10. doi: 10.3389/fncom.2020.00010 (PMC7041417; doi:10.3389/fncom.2020.00010)
Supplement: Supplementary file 1 [file Data_Sheet_1.PDF]

Supplementary Material

Table 1: Results of expert-radiologist grading on local dataset. Score 0 referred to the poor segmentation and 5 for the most accurate delineating of the tumor parts from the healthy tissues. The evaluation is carried on 40 patients.

Score	Number of patients	Average Score	Median Score
**0**	0	4.1	4
**1**	2		
**2**	1		
**3**	2		
**4**	21		
**5**	14		

Table 2: Performance with various hyper-parameters on BraTS18 validation dataset.

Method	Dice Similarity Score		
	**ET**	**WT**	**TC**
Patch Size: 32×32×32, Feature Maps: 16,32,64 Loss: Dice	0.63	0.79	0.71
Patch Size: 32×32×32, Feature Maps: 16,32,64 Loss: Cross-Entropy	0.61	0.72	0.62
Patch Size: 32×32×32, Feature Maps: 48,96,192 Loss: Dice	0.67	0. 78	0.69
Patch Size: 64×64×64, Feature Maps: 16,32,64 Loss: Dice	0. 71	0.80	0.76
Patch Size: 64×64×64, Feature Maps: 16,32,64 Loss: Cross-Entropy	0. 68	0. 79	0.72
**Patch Size: 64×64×64, Feature Maps: 48,96,192 Loss: Dice**	**0.75**	**0.87**	**0.82**

MRI Protocol

The Machine: 3-tesla system (3T HDXE-, GE Healthcare) at will be used in all cases.

Plain sequences: T2 W Cube and Isotropic 3D T1w and FLAIR sequences will be performed. This replaces several slice-by-slice, plane-after-plane 2D acquisitions with a single 3D volume scan. So anisotropic image will give every bit of information that is needed for evaluating even the smallest lesions. This will be easily be reconstructed high-definition, sub-millimeter-resolution images from a single 3Dacquisition into any desired plane–without gaps and with the same resolution as the original plane in which it was acquired. And because the entire 3D story will be captured in one time-efficient scan, it saves exam time.

Susceptibility weighted imaging (SWI), originally called BOLD venographic imaging, uses a type of contrast in magnetic resonance imaging (MRI) different from traditional spin density, T1, or T2 imaging. SWI uses a fully flow compensated, long echo, gradient echo (GRE) scan to acquire images. The enhanced sensitivity of SWI to venous blood and blood products due to their differences in susceptibility compared too normal tissue leads to better contrast in detecting tumor boundaries and tumor hemorrhage.

Pre and post contrast BRAVO: Brain Volume imaging is a high-resolution 3D imaging technique designed to produce heavily T1-weighted isotropic images of the brain. BRAVO deploys 3D IR-prepared FSPGR acquisition to produce isotropic T1-weighted volumes. The center of k-space is oversampled and will serve as the calibration data for the parallel-imaging reconstruction. Innovative 2D ARC parallel imaging will help reduce scan time and will minimize parallel imaging artifacts.

Volume assessment:1D tumor size will be the sum of the longest diameters (measured with calipers) from each measurable contrast-enhanced tumor on the post gadolinium T1-weighted axial or sagittal images. 2D measurements will be the sum of the products of the largest diameters and their maximum perpendicular diameters in the same plane from each measurable contrast-enhanced tumor on the post gadolinium T1-weighted scans. 3D measurements will be performed on a GE Medical Systems Windows Advantage Workstation. The contrast-enhanced tumor on digitized postcontrast T1-weightedimages as well as T2 signal abnormality (as deemed appropriate by the clinician and radiologist in conjunction) will be outlined in three planes with a paintbrush technique. The tumor volume will be calculated by the workstation from these user-defined tumor images.
